# Optimizing Prednisolone Loading into Distiller’s Dried Grain Kafirin Microparticles, and In vitro Release for Oral Delivery

**DOI:** 10.3390/pharmaceutics9020017

**Published:** 2017-05-19

**Authors:** Esther T. L. Lau, Stuart K. Johnson, Barbara A. Williams, Deirdre Mikkelsen, Elizabeth McCourt, Roger A. Stanley, Ram Mereddy, Peter J. Halley, Kathryn J. Steadman

**Affiliations:** 1 School of Pharmacy, University of Queensland, 4072 Brisbane, Australia; et.lau@qut.edu.au (E.T.L.L.); elizabeth.mccourt@hdr.qut.edu.au (E.M.); 2 School of Clinical Sciences, Queensland University of Technology, 4000 Brisbane, Australia; 3 Nutrition, Dietetics and Food Technology, School of Public Health, Faculty of Health Sciences, Curtin University, 6845 Perth, Australia; s.johnson@curtin.edu.au; 4 Centre for Nutrition and Food Sciences, Queensland Alliance for Agriculture and Food Innovation, University of Queensland, 4072 Brisbane, Australia; b.williams@uq.edu.au (B.A.W.); d.mikkelsen@uq.edu.au (D.M.); roger.stanley@utas.edu.au (R.A.S.); ram.mereddy@daf.qld.gov.au (R.M.); 5 ARC Centre of Excellence in Plant Cell Walls, University of Queensland, 4072 Brisbane, Australia; 6 Innovative Food Technologies, Department of Agriculture and Fisheries, 4108 Brisbane, Australia; 7 Centre for Food Innovation, University of Tasmania, 7001 Hobart, Australia; 8 Australian Institute for Bioengineering and Nanotechnology, University of Queensland, 4072 Brisbane, Australia; p.halley@uq.edu.au; 9 School of Chemical Engineering, University of Queensland, 4072 Brisbane, Australia

**Keywords:** kafirin, distiller’s dried grains with solubles, microparticles, response surface methodology, simulated gastrointestinal conditions, colonic delivery

## Abstract

Kafirin microparticles have potential as colon-targeted delivery systems because of their ability to protect encapsulated material from digestive processes of the upper gastrointestinal tract (GIT). The aim was to optimize prednisolone loading into kafirin microparticles, and investigate their potential as an oral delivery system. Response surface methodology (RSM) was used to predict the optimal formulation of prednisolone loaded microparticles. Prednisolone release from the microparticles was measured in simulated conditions of the GIT. The RSM models were inadequate for predicting the relationship between starting quantities of kafirin and prednisolone, and prednisolone loading into microparticles. Compared to prednisolone released in the simulated gastric and small intestinal conditions, no additional drug release was observed in simulated colonic conditions. Hence, more insight into factors affecting drug loading into kafirin microparticles is required to improve the robustness of the RSM model. This present method of formulating prednisolone-loaded kafirin microparticles is unlikely to offer clinical benefits over commercially available dosage forms. Nevertheless, the overall amount of prednisolone released from the kafirin microparticles in conditions simulating the human GIT demonstrates their ability to prevent the release of entrapped core material. Further work developing the formulation methods may result in a delivery system that targets the lower GIT.

## 1. Introduction

Kafirin microparticles have been investigated for their potential as an oral delivery system. Kafirins are storage proteins of the sorghum grain, and their characteristic hydrophobic and poorly digestible nature [1,2,3] has been identified as a potential delivery system that can target or delay the release of medications. For instance, approximately 20–25% of the prednisolone loaded into microparticles made using kafirin extracted from whole grain sorghum, was released over seven hours in simulated gastric and small intestinal conditions. These results suggest the kafirin microparticles have potential as a colon-targeted delivery system since they can protect the majority of the encapsulated material from being released in the upper GIT [4]. One of the reasons for the low digestibility of the kafirin protein is thought to be due to its formation of multiple disulfide bond crosslinks, which protects against hydrolysis by digestive enzymes [5,6,7]. Previous work on polysaccharides and pro-drugs have shown that delivery systems targeting the microbes of the colon holds promise as a reliable colon-targeted drug delivery system [8,9,10,11,12,13]. Given the presence of the colonic (anaerobic) bacterial microbiota, and the resulting low redox, it is hypothesized that these disulfide bonds may be reduced (cleaved) upon entry to the colon, thereby releasing the encapsulated drug.

To be commercially viable as a drug delivery system, wastage of material must be minimized during the process of formulating these microparticles. As the factors involved in influencing prednisolone loading into the kafirin microparticles are not yet well understood, it is difficult to manipulate and optimize the amount of drug (core material) that is loaded. Response surface methodology (RSM) is a well-established statistical modeling method that has previously been used in the pharmaceutical industry to model and optimize formulations of drug delivery systems. Some examples include tamsulosin pellets [14], indomethacin pellets [15], zein microcapsules loaded with flax oil [16], chitosan microparticles loaded with felodipine [17], Eudragit® microparticles loaded with captopril [18], controlled release mesalazine tablets [19], and controlled release atenolol tablets [20] for oral delivery. RSM has also been used to model the formulation of diclofenac and curcumin gels for topical delivery [21]. RSM involves identifying factors that contribute to the desired outcomes. The interactions between the factors that affect the outcome are mapped so that optimum conditions can be established. RSM uses statistical modeling with regression equations to describe the relationship between these factors and the outcomes. From these models, an optimized combination of factors may be determined to generate a desired outcome. For instance, in designing formulations for drug delivery systems, the aim would be to minimize the amount of material used or wasted, whilst maximizing the amount of drug loaded [22].

Based on preliminary work, three variables appear to have an effect on the amount of prednisolone loaded into the microparticles: the starting quantities of kafirin; the starting quantities of prednisolone; and the inclusion of sodium chloride (NaCl) [23]. In this work, RSM will be used to build a statistical model that can be used to predict the combination of independent variables which are required to achieve any level of prednisolone loading and loading efficiency, i.e., the dependent variables. This will allow optimization of the formulation to produce kafirin microparticles loaded with prednisolone. The kafirin formulation with the optimal prednisolone loading will then be tested for their potential as an oral delivery system in conditions simulating the stomach and small intestine, and in the colon. The particle size and thermodynamic stability of the prednisolone-loaded microparticles will also be measured over a period of six months.

## 2. Materials and Methods

### 2.1. Kafirin Source and Extraction, and Formulating Kafirin Microparticles

Distiller’s dried grains (DDG) kafirin was extracted from the protein-rich waste stream following biofuel production (i.e., DDGS) with red grain sorghum at a sorghum refinery (Dalby Bio-refinery, Dalby, Australia) [23]. Microparticles were formulated according to the method reported for loading prednisolone into kafirin microparticles [23], which was adapted from formulating zein microparticles [24]. A solution of NaCl was used to form the prednisolone-loaded microparticles by inducing phase separation in a prednisolone-ethanol solution. The microparticles were freeze-dried overnight before being analyzed for prednisolone loading, and loading efficiency into the microparticles using HPLC [23,24]. Reagents were of analytical and HPLC grades.

### 2.2. Response Surface Methodology

The independent variables, kafirin protein (*X*_1_) and the amount of prednisolone (*X*_2_), were investigated for their effects on the dependent variables, prednisolone loading (*Y*_1_) and loading efficiency (*Y*_2_) (Table 1). Drug loading was not significantly different across the range of NaCl concentrations investigated (0.01–5 M) [23], so the amount of NaCl used in the formulation was kept constant at 8 mL of 0.1 M.

A two-factor, three-level central composite design was used, with five replicates at the center point, generating a total of 13 runs. The levels of the independent variables investigated were 5–20 mg/mL kafirin (i.e., 100–400 mg), and 5–17.5 mg/mL of prednisolone (i.e., 100–350 mg), with each experimental formulation made in triplicate. These ranges were determined based on the preliminary work on loading prednisolone into kafirin microparticles [23], in conjunction with the work on zein microparticles loaded with prednisolone [25]. The variable effects were modelled using the equation for two process variables [15,16,26,27,28]:

*y* = β_0_ + β_1_*X*_1_ + β_2_*X*_2_ + β_11_*X*_1_^2^ + β_22_*X*_2_^2^ + β_12_*X*_1_*X*_2_ + *ε* (1)

where *y* is the dependent variable (or response), β_0_ is the intercept, β_1_ and β_2_ are the regression coefficients of the linear order effect, β_11_ and β_22_ are the quadratic order effect, β_12_ is the linear interaction order effect, and *ε* is the experimental error term [16,19,26,28]. Design-Expert 8.0.6 (Stat-Ease, Inc., MN, USA) was used to model the data. The level of significance was set at *p* < 0.05, and r^2^ and lack of fit were used to gauge the adequacy of the model [29]. Response surface plots and contour plots were generated using the polynomial equations. The surface plots allowed the relationship between the independent variables and responses to be visualized. These were used to predict the levels of independent variables required to produce a defined response. The contour plots provided a continuous predictive tool to predict the response given any combination of independent variables.

### 2.3. Optimization Data Analysis and Validation of Optimization Model

The combination of independent variables required to produce an optimal response based on a set of imposed constraints can be calculated with the model generated using RSM. Prednisolone loading is a measure of the proportion of prednisolone in the microparticles, so a higher value is more desirable. Similarly, a higher loading efficiency is more desirable as it is a measure of the amount of prednisolone loaded, as a percentage of the amount of prednisolone initially added. Thus, the goals of the optimization prediction were to maximize both prednisolone loading and loading efficiency. During the calculation of the optimization statistics, equal importance was assigned to each variable and both responses, as it was equally important for both prednisolone loading and loading efficiency to be maximized, whilst minimal or reasonable quantities of kafirin and prednisolone were used. Design-Expert 8.0.6 (Stat-Ease Inc, Minneapolis, MN, USA) was used to plot the optimal combination of variables required to generate the desired response of maximized drug loading and loading efficiency from the RSM models. To obtain an acceptable prediction of the optimal combination of kafirin and prednisolone, the minimal level of the responses used was 10% for drug loading and 15% for loading efficiency. The relationship between the variables and both responses were mapped on a single contour plot, essentially producing an overlay of the contour plots for prednisolone loading and loading efficiency, providing a visual representation of the optimized outcome. The predicted optimal and non-optimal combinations were reformulated in triplicate to verify the model. A ′non-optimal′ combination of 200 mg kafirin and 200 mg prednisolone was arbitrarily selected as it was not used originally in the development of the model, and because it is within the boundaries of the range of independent variables used in this RSM study (i.e., 100–350 mg prednisolone, and 100–400 mg kafirin).

The error between the values of the responses predicted by the RSM model, and the experimentally determined values of the responses for the ′optimal′ and ′non-optimal′ formulations were calculated as follows:

(2) $$\mathrm{Predicted}\;\mathrm{error}\;\left(\%\right)=\frac{\mathrm{Observed}\;\mathrm{value}-\mathrm{Predicted}\;\mathrm{value}}{\mathrm{Predicted}\;\mathrm{value}}\times 100$$

### 2.4. Scanning Electron Microscopy

The freeze-dried kafirin microparticle samples were mounted onto carbon tabs and coated with 10 nm of platinum using a sputter coater (Baltec MED 020, Leica Microsystems, Wetzlar, Germany). The samples were then viewed at 5 kV and a working distance of 8 mm using a field emission scanning electron microscope JSM 6300F (JEOL, Tokyo, Japan) [4,23,25].

### 2.5. Particle Size Distribution

Freeze-dried microparticles (50 mg) dispersed in 50 mL of 40% (*v*/*v*) ethanol were analyzed by laser diffraction using a Mastersizer 2000E (Malvern Instruments, Malvern, UK) and a small volume sample dispersion unit (Malvern Instruments, Malvern, UK). The refractive index used for kafirin was 1.45 and 1.33 for 40% (*v*/*v*) ethanol. The obscuration range was between 10% and 20%, with a residual of less than 1%. Particle size was measured in duplicate for each sample [25].

### 2.6. Stability Testing

Stability studies were conducted on the prednisolone loaded kafirin microparticles at 4.5 °C and 25°C/60% relative humidity, and under accelerated conditions at 40°C/60% relative humidity for 6, 12 and 24 weeks [30]. Aliquots (10 mg) of the optimal microparticle formulation identified using RSM were weighed into low protein binding centrifuge tubes, capped, and then wrapped in foil (three replicates for each storage condition and temperature). Microparticles formulated without kafirin were used as the drug control. At each time-point, three aliquots of each microparticle formulation were removed and the amount of drug leakage as measured by the amount of prednisolone washed out was quantified using HPLC [4,23,25].

### 2.7. *In vitro* Drug Release

#### 2.7.1 Simulated Gastric and Small Intestinal Conditions

Prednisolone release from the optimal microparticle formulation identified using RSM were measured in simulated gastric and small intestinal conditions. The United States Pharmacopeia (USP) dissolution Apparatus 2 fitted with a small volume vessel (100 mL) was used [31]. The medium was made according to the British Pharmacopoeia (BP) [32], for a test using increasing pH to simulate the stomach and small intestine for 7 h. Drug release was also measured using the same medium in the presence of enzymes, where pepsin (0.32 g, 0.7 FIP-U/mg; AppliChem, Darmstadt, Germany) was added to the medium at 0 h, and pancreatin (1 g, 23664 FIP-U/g; AppliChem, Darmstadt, Germany) was added at 1 h. In a separate test, prednisolone release from the kafirin microparticles was also measured in simulated small intestinal conditions with pancreatin in the presence of bile salts for 4 h. Simulated intestinal fluid with pancreatin was made according to the British Pharmacopoeia [32], then 8 mg/mL of bile salts (Chem-Supply Pty Ltd, Gillman, Australia), equivalent to c. 15 mM, was added as it is purportedly similar to the bile salt concentration present in human intestinal fluids in a fed state [33,34,35]. Drug release from approximately 30 mg of microparticles containing 5 mg of prednisolone was compared with that of a commercially available 5 mg prednisolone tablet (Panafcortelone^TM^, Aspen Pharmacare, St Leonards, Australia). At regular time intervals, samples were retrieved and analyzed using HPLC [4,23,25].

#### 2.7.2. Simulated Colonic Conditions

An In vitro fermentation method using pig feces was used to simulate the conditions of the colon [36], as a pig model of digestion is appropriate for mimicking human colonic function [37]. Furthermore, it is convenient to use porcine fecal inoculum due to consistency between the pigs, and also because the diet of the pigs can be controlled. This means that measures can be taken to ensure that the microbes have not been pre-exposed to the substrates tested, which would otherwise allow the microbial enzymes to adapt [37].

##### Fecal Inoculum

Feces were collected per-rectum from five pigs that were offered a ′control diet′, which was based on highly digestible corn starch and fishmeal, for at least 10 days. The feces were placed into a pre-warmed vacuum flask that had been flushed with CO_2_ for transfer back to the laboratory. The feces were combined and diluted in a ratio of 1:5 using sterile saline (0.9% *w*/*v* NaCl) pre-warmed to 39 °C. This mixture was mixed using a hand-held blender for 1 min, strained through four layers of muslin cloth, then stirred constantly and flushed with a continuous stream of CO_2_, before being added (5 mL) to the substrates and medium. For the autoclaved fecal inoculum, a sample of the blended and strained mixture was autoclaved at 121 °C for 15 min, before 5 mL was added to the substrates and medium as a control.

##### Substrates and Medium

The medium was prepared using an In vitro batch culture method consisting of the basal solution (68 mL), reducing agent (1 mL), bicarbonate solution (1 mL), and vitamin-phosphate solution (4 mL) [36]. Pre-weighed glucose was added to provide a source of energy (carbohydrate) for the microbes.

Table 2 provides a summary of the substrates used for fermentation. Three replicates of kafirin microparticles loaded with prednisolone were analyzed (Table 2; substrates 1–3). Six replicates of each substrate (c. 30 mg) were then weighed into serum bottles for fermentation. One blank and four controls were used, of which three contained no prednisolone (Table 2; substrates 4–6). Commercially available 5 mg prednisolone tablets were included as positive controls, with substrate 8 (the autoclaved fecal inoculum) used to assess any impact the fecal matter had on prednisolone detection.

Three replicates from each substrate were used for gas measurements. These bottles were randomized on a single tray and fermented at 39°C. Samples (2 mL) were removed from the remaining three replicates at regular time intervals, to quantify the amount of prednisolone present at –16, 0, 2, 4, 8, 12, 19 and 24 h. Each bottle was gently swirled to achieve a homogenous mixture before the samples were removed. The –16 h samples were taken one hour after the medium had been added to all the bottles at –17 h, while the 0 h samples were when the fecal inoculum was added to the bottles. The 2 mL samples were centrifuged at 15,000 rpm at 4 °C for 5 min. The resulting supernatant was filtered through 0.45 µm Durapore filter paper before being analyzed by HPLC [4,23,25].

### 2.8. Statistical Analysis

One-way ANOVA with a Bonferroni post-hoc test were used to compare the percentage of prednisolone released after one hour of the dissolution study from the kafirin microparticles in the presence and absence of enzymes. GraphPad Prism 5.04 (GraphPad Software, San Diego, CA, USA) was used with the level of significance set as *p* < 0.05.

## 3. Results

### 3.1. Response Surface Methdology

At the various concentrations of kafirin and prednisolone used, prednisolone loading ranged from 2.13% to 15.57%, while loading efficiency ranged from 5.26% to 36.92%. The model generated using the regression coefficients relating the dependent variables to the independent variables in coded values were:

Prednisolone loading (*Y*_1_) = +7.25 – 0.21*X*_1_ + 3.37*X*_2_ (3)

Loading efficiency (*Y*_2_) = +14.67 + 5.98*X*_1_ – 1.38 *X*_2_ + 5.99*X*_2_^2^ (4)

**Figure 1:**
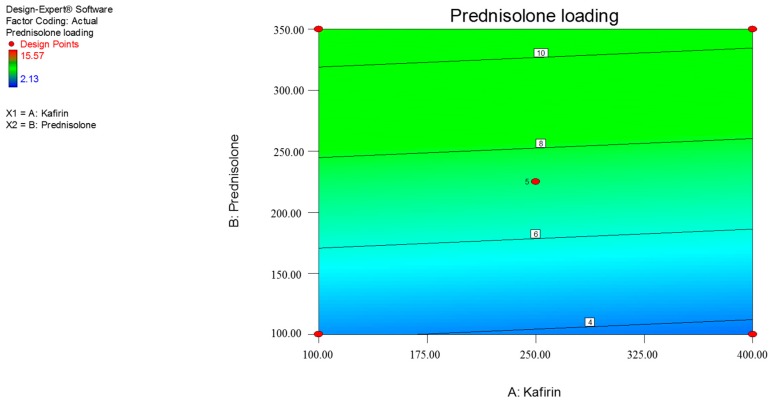
Contour plot showing the effects of kafirin and prednisolone quantities (mg) on (*Y*_1_) prednisolone loading (%). The numbers in the small squares refer to the response value.

**Figure 2:**
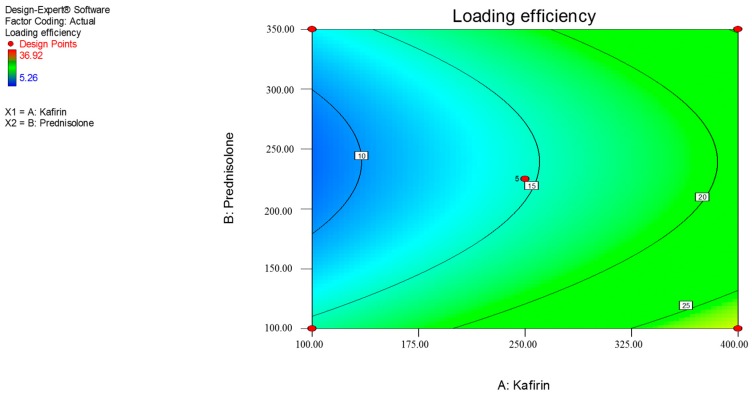
Contour plot showing the effects of kafirin and prednisolone quantities (mg) on (*Y*_2_) loading efficiency (%). The numbers in the small squares refer to the response value.

This predicts that a higher starting quantity of prednisolone will produce higher drug loading. Similarly, high loading efficiency may be achieved with larger quantities of kafirin. Prednisolone loading (%) significantly increased with increasing quantities of prednisolone, while there was a negative trend with kafirin quantity (Figure 1, Equation (3)); though this was not statistically significant (Table 3). Similarly, the model predicts that loading efficiency (%) significantly increases with increasing kafirin quantities (mg), while the effect of prednisolone quantities (mg) on loading efficiency demonstrated a strong trend at the quadratic level (*p* = 0.0522) (Figure 2, Equation (4) and Table 3).

Overall, the model (linear) for prednisolone loading (Equation (3)) was significant (*p* = 0.0174), with an insignificant lack of fit (*p* = 0.0882) supporting the adequacy of the model. However, the r^2^ of the model was only 55.54%, which indicates that the model is not well correlated with the data gathered in our experiments, reducing its adequacy for predictive purposes. The loading efficiency model (quadratic) shows a strong trend towards significance (*p* = 0.0573). The lack of fit for the quadratic loading efficiency model is also significant (*p* = 0.0502) and has a r^2^ of 54.85%. Ideally, an adequate model would be statistically significant, have an insignificant lack of fit, and r^2^ of at least 80% [29]. Hence, the RSM models generated in this study lack adequacy for modeling purposes. Nevertheless, these RSM models are still useful in identifying trends between the independent and dependent variables.

### 3.2. Optimization Data Analysis and Validation of Optimization Model and Thermodynamic Stability

**Figure 3:**
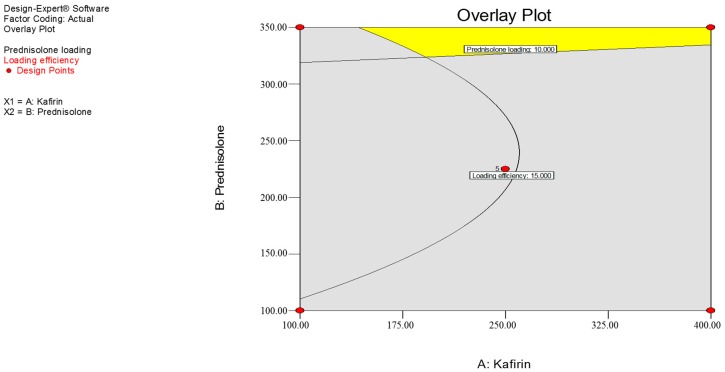
Overlay of the contour plots to visualize the quantities of kafirin (*X*_1_) and prednisolone (*X*_2_) required to maximize prednisolone loading (*X*_1_), and loading efficiency (*X*_2_). The numbers in the small squares refer to the response value.

The predicted optimal combination can be visualized on the overlay of the prednisolone loading and loading efficiency contour plots (Figure 3). The yellow panel on the top right-hand corner of the overlay plot shows the optimal zone where the constraints are met according to this RSM model. As such, the RSM model predicted that the optimal combination of kafirin and prednisolone for maximized drug loading and loading efficiency was 400 mg kafirin and 350 mg prednisolone (i.e., one of the experimental combinations). The predicted and observed values for both prednisolone loading and loading efficiency had an error of c. 20–30%, so the predicted value was lower than that observed for the predicted optimal combination (Table 4). Conversely, the non-optimal combination had c. –40% predicted error, with the predicted value being higher than the observed value for prednisolone loading and loading efficiency. Additionally, the amount of prednisolone washed out (i.e., drug leakage) from the optimal combination of kafirin and prednisolone that were stored at the different temperatures was not significantly different over the six month period.

### 3.3. Scanning Electron Micrographs

**Figure 4:**
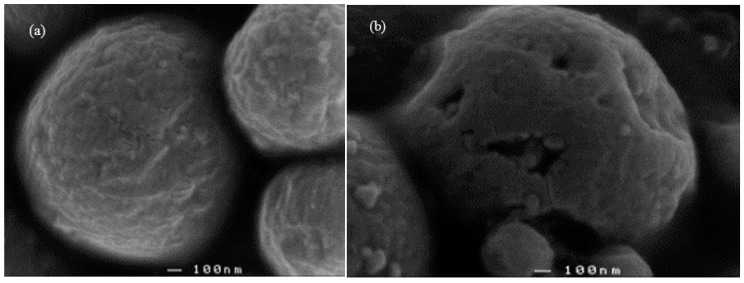
Scanning electron micrograph of microparticles formulated using 400 mg distiller’s dried grains (DDG) kafirin (**a**), and 350 mg prednisolone with 400 mg DDG kafirin (**b**) at 50,000×.

Multiple scanning electron micrographs were taken, with two representative micrographs presented here. Scanning electron micrographs (Figure 4a,b) confirmed the presence of round particles that were varied in size. The surface structure of the microparticles was irregular and crinkled, though the prednisolone loaded microparticles were generally more crenated. The prednisolone loaded microparticles also appeared to have pores or pits on the surface.

### 3.4. Particle Size Distribution

The empty and prednisolone loaded microparticles formulated using DDG kafirin both had a wide size distribution, with 90% being less than c. 34 µm (Table 5). The surface weighted mean (D(3,2)) and the volume weighted mean (D(4,3)) also indicated that the microparticles were similar in size, regardless of whether any prednisolone was loaded.

### 3.5. *In vitro* Drug Release

#### 3.5.1. Simulated Stomach and Small Intestinal Conditions

**Figure 5:**
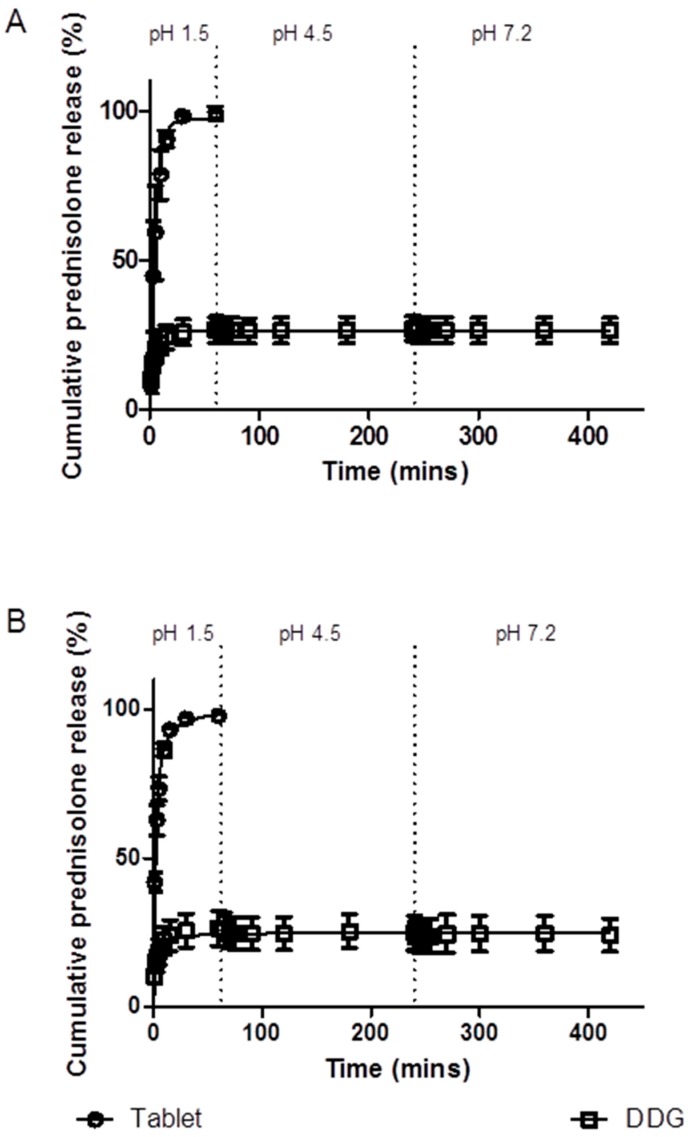
In vitro drug release profile of a commercially available 5 mg prednisolone tablet compared to prednisolone release from the kafirin microparticles containing an equivalent of 5 mg prednisolone. Prednisolone release was measured in conditions simulating the stomach for 1 h, followed by the small intestine for 6 h, (**a**) without enzymes, and (**b**) in presence of the enzymes pepsin and pancreatin, respectively. Values are mean ± SEM (*n* = 3).

**Figure 6:**
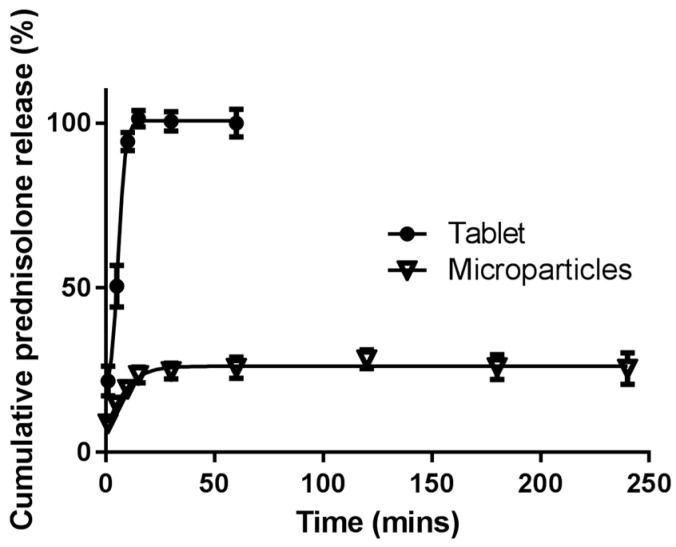
In vitro drug release profile of a commercially available 5 mg prednisolone tablet compared to drug release from kafirin microparticles containing an equivalent of 5 mg prednisolone. Prednisolone release was measured in conditions simulating the small intestine using simulated intestinal fluid, pancreatin, and bile salt for 4 h. Values are mean ± SEM (*n* = 3).

In the conditions simulating the stomach and small intestine, the extent of prednisolone release from the commercial tablet was higher than that from the microparticles. Approximately 100% of the prednisolone was released from the tablets within 30 min, whereas only approximately 15–25% of prednisolone was released from the microparticles. In both the commercial tablet and the microparticles, the extent of drug release had peaked after 30 min, before plateauing out for the remainder of the 7 h (Figure 5). The presence of pepsin and pancreatin did not impact upon drug release; 26.60% ± 4.37% in the presence of enzymes versus 26.33% ± 5.93% in the absence of enzymes (mean ± SEM) (Figure 5). Prednisolone release from both the kafirin microparticles and commercial tablets in the presence of bile salts (Figure 6) mimicked that of the 7 h drug release study (Figure 5). Approximately 100% of the prednisolone was released from the tablets within 30 min, while 25.67% ± 3.2% (mean ± SEM) of the prednisolone was released from the microparticles at 1 h, before plateauing out for the remainder of the 4 h.

#### 3.5.2. Simulated Colonic Conditions

**Figure 7:**
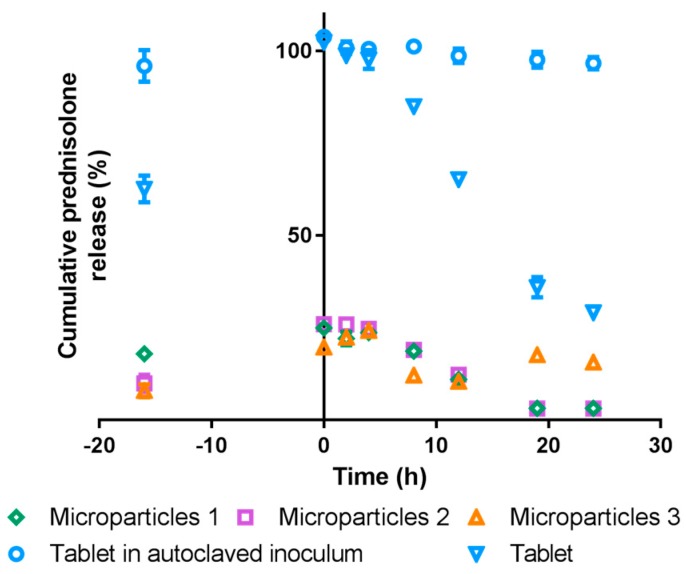
In vitro drug release profile of a commercially available 5 mg prednisolone tablet, compared to drug release from kafirin microparticles containing an equivalent of 5 mg prednisolone, in conditions simulating the colon. Medium was added to the substrates (0.5 g glucose with microparticles or tablet; see Table 2) at –16 h, and fecal inoculum was added at 0 h. A tablet incubated in autoclaved inoculum served as a control (Control 4 in Table 2). Values are mean ± SEM (*n* = 3).

While 100% of the prednisolone from the tablet could be measured in the simulated gastric and intestinal conditions, only a maximum of approximately 85% could be detected in the simulated colonic testing when in the presence of any fecal inoculum. In previous studies, if fecal inoculum was not included with the medium, then 100% of the prednisolone could be recovered using the 5 mg commercial prednisolone tablet. This suggests that in this present study, 15% of the prednisolone released could not be detected due to possible interactions with the fecal inoculum. Hence, as the maximum amount that was detected for a tablet of 5 mg prednisolone was 4.25 mg, the prednisolone release profile was adjusted to account for this (Figure 7).

Within 60 min after the medium was added to the substrates (–16 h), prednisolone was detected (Figure 7). At 0 h, i.e., the point at which the fecal inoculum was added, 100% of the prednisolone from the tablets had been released, while approximately 25% of the prednisolone had been released from the microparticles. When incubated with autoclaved fecal inoculum, release from the 5 mg tablet remained at approximately 100% for the entire 24 h incubation, but with standard fecal inoculum (i.e., not autoclaved) the prednisolone decreased after 4 h to approximately 65% at 12 h and 30% at 24 h. Kafirin microparticles loaded with prednisolone responded in a similar manner to the commercial tablet when incubated with standard fecal inoculum, in that the amount of prednisolone detected was maximal (25%) at 0 h, but then declined from 4 h incubation to approximately 10% at 12 h. The amount of prednisolone detected continued to decrease, such that at 19 h and 24 h there was a negligible amount (Figure 7). No prednisolone was detected in the blank and control substrates.

## 4. Discussion

To be feasible as an oral drug delivery system, the amount of kafirin microparticles required to deliver a clinically relevant dose must be thermodynamically stable and realistic for oral intake [4,23]. The RSM model described a significant linear relationship between prednisolone concentration and prednisolone loading into the kafirin microparticles. Increasing the starting concentration of prednisolone was associated with an increase in the amount of prednisolone retained, as was also observed with the surface binding [23], and with previous work using zein microparticles loaded with prednisolone [25], ivermectin [38], and gitoxin [39]. The starting concentration of kafirin was the only significant term described in the quadratic model of loading efficiency, but only demonstrated a trend towards significance. Higher loading efficiency was achieved with higher kafirin concentrations. This was also consistent with prednisolone [25], ivermectin [38], and gitoxin [39] loading into zein microparticles, tadalafil loading into microporous silica [40], and captopril loading into Eudragit^®^ and Methocel^®^ microparticles [18]. This was not unexpected, as the greater availability of coating material may have allowed more core material to become encapsulated.

However, the model also predicted that regardless of the amount of kafirin, small amounts of prednisolone (e.g., 100 mg) would produce higher loading efficiency than larger amounts (e.g., 350 mg), with intermediate quantities of prednisolone producing the lowest loading efficiency. This relationship is not consistent with the trends noted for zein-prednisolone microparticles [25], nor those in the literature [16,38,39], which suggest that larger ratios of coating material to core material produce higher loading efficiencies. This discrepancy might be explained by the inadequacy of the generated model. The large error noted between the predicted and observed values (Table 4) indicates the RSM models are inadequate for accurately predicting prednisolone loading or loading efficiency. The BP states that for prednisolone tablets, the content must be 90.0–110.0% of the stated amount [41]. As such, the current models generated would not be acceptable tools for predicting formulations for drug delivery purposes.

RSM is a tool that has long been used for statistical modeling. The inadequacy of the models suggests that the data used to generate the models is lacking in robustness. During the microparticle formulation process using 70% (*v*/*v*) ethanol, experimental variables that were controllable—such as temperature, mixing times, intensity of vortex mixing, ethanol and NaCl concentration—were kept constant, with only the kafirin and prednisolone quantities being varied. Of these two variables, the quantities of prednisolone used readily dissolved in 70% ethanol at ambient temperature. However, even at 70 °C, only approximately 90% of the kafirin protein could be dissolved in 70% ethanol, and small gelled pellets of insoluble residues were formed as a result of a tendency for kafirin to gelate when dissolved in aqueous ethanol [42]. The formation of these pellets could not be controlled during the microparticle formulation process. Thus, inconsistencies in the amount of kafirin dissolved and gelled pellets formed would cause unintended variability in the amount of prednisolone encapsulated, negatively impacting upon the robustness of the model. As such, an alternate solvent such as glacial acetic acid at 25 °C, which dissolved significantly more kafirin protein (c. 95%) without the formation of gelled pellets [42], may reduce variability in the amount of prednisolone encapsulated to help improve the robustness of the model. Additionally, the effect of NaCl on microparticles formation was perhaps two-fold. Aside from its charge shielding effect, it may also have had an additional ′salting out′ effect [43] which promoted kafirin to precipitate out of solution. It is likely that prednisolone was loaded into the kafirin microparticles when both were co-precipitated, with prednisolone and kafirin first aggregating together into nanoparticles, then precipitating with other nanoparticle aggregations to form the resulting microparticles. This would account for the varied sizes, and the crenated and layered appearance of the microparticles (Figure 4). Therefore, investigating alternate methods for formulating the microparticles may also contribute to the robustness of the model, and also formulating microparticles that are more homogenous in size.

The initial burst release of prednisolone from the microparticles, followed by a slower sustained release which plateaus, in simulated gastric and small intestinal conditions, is consistent with previous work on kafirin and zein microparticles [4,25]. The lack of prednisolone released after the initial burst release may be due to the inherent nature of the kafirin protein. The hydrophobic nature of both the kafirin protein and prednisolone (log*P* = 1.62 [44]) does not favor the release of prednisolone into an aqueous environment. This lack of further prednisolone release may be enhanced by the hydrophobic interactions between kafirin and prednisolone. These hydrophobic interactions may also have prevented the change in protein conformation that was observed with a pH change, resulting in further antioxidant activity when catechins and condensed tannins were encapsulated in kafirin microparticles [1]. The hydrophobic interactions promoted by the addition of NaCl to achieve prednisolone loading, along with the innate low digestibility of kafirin [2,3], may have prevented the release of prednisolone under the simulated stomach and small intestinal conditions. Digestive enzymes and bile salts did not affect prednisolone release from the microparticles, further supporting the postulation that the encapsulated drug is protected from the digestive processes of the upper GIT.

In simulated conditions of the colon, prednisolone was detected in the –16 h incubation time samples. The presence of prednisolone in the medium before the addition of any fecal inoculum is consistent with the substrates being in contact with an aqueous liquid. This was observed with the prednisolone release in simulated conditions of the stomach and small intestine, since enzymes did not affect the amount of prednisolone released (Figure 5). Less prednisolone was detected at –16 h incubation time samples in the simulated colonic conditions (60 min after the medium was added to the substrates), compared to the 1 h samples from simulated gastric conditions (Figure 5 and Figure 7). This may be explained by the differences in the agitation method. In the simulated gastric conditions, the mixture was under constant agitation at 100 rpm, while the samples in the simulated colonic conditions were gently swirled before a sample was removed. Less vigorous agitation of samples in the simulated colonic conditions would account for the slower rate of release observed initially. Nevertheless, the amount of prednisolone at 0 h in the simulated colonic conditions (Figure 7) is similar to the total amount released over 7 h in simulated gastric and intestinal conditions (Figure 5). This can be likened to the microparticles passing through the stomach and small intestine before reaching the colon (i.e., at 0 h).

The subsequent decrease in the amount of prednisolone quantified is consistent with the microbes reducing the ′A ring′ of the prednisolone structure, rendering it undetectable at 254 nm [45]. This effect of the microbes on prednisolone was also observed when prednisolone was incubated in 10% rat cecal contents, whereby 50% of the prednisolone added was undetectable after 4 h incubation [45]. As the inoculum concentration affects the rate of prednisolone breakdown [45], this would explain the faster decline in the 10% rat cecal contents compared to our c. 6% of porcine feces. Furthermore, the differences in the rate of decline may also be attributed to the innate differences in the sources of inoculum (rat cecal contents versus porcine feces), and the different diets and environments to which these animals were exposed.

It is evident that introducing the kafirin microparticles to a reducing environment did not result in further drug release, as the maximum amount of prednisolone was quantified at 0 h incubation time for all substrates. It appeared that the rate of prednisolone reduction was slower with microparticles compared to the tablet. The faster rate of prednisolone reduction with the tablet may also be explained by the larger quantity of prednisolone in the medium when the fecal inoculum was added (5 mg for the tablet versus c. 1 mg for the microparticles). Additionally, the microbes incubated with the tablets were more active, as was observed with the readings of the gas released by the microbes during fermentation (data not shown), possibly due to the presence of fermentable excipients (maize starch and lactose) in the tablet [46]. In contrast, kafirin appeared to inhibit the fermentation activity of the microbes. In combination, these factors may account for the faster rate of prednisolone reduction with the tablet. An alternate explanation for the different rates of decreasing prednisolone is that the effects of prednisolone degradation were being counteracted by additional prednisolone release from the microparticles. Nevertheless, the rate of prednisolone degradation was still faster than the rate of prednisolone being released, and reduction of the ′A ring′ which causes prednisolone to be undetectable at 254 nm also causes a decrease in the anti-inflammatory effects of prednisolone [45]. In light of this, even if additional prednisolone was released, it is unlikely to provide any clinical benefits.

## 5. Conclusions

The RSM models generated were inadequate for accurately predicting the relationship between the starting quantities of kafirin and prednisolone, and prednisolone loading and loading efficiency. The robustness of the model needs to be improved with better data before it can be used to predict optimal combinations to formulate kafirin microparticles loaded with prednisolone for use as an oral drug delivery system. The lack of further prednisolone release in simulated colonic conditions suggests that delivering prednisolone with kafirin microparticles formulated using this present method of phase separation is unlikely to offer any clinical benefits over the currently available commercial dosage forms. Nevertheless, the overall amount of prednisolone released from the kafirin microparticles in conditions simulating the human digestive system demonstrates their ability to prevent the release of entrapped core material. Further work developing the kafirin formulation and drug loading methods may result in a delivery system that targets the lower GIT.

## Figures and Tables

**Table 1:** Variables in the central composite design.

**Real Independent Variables**	**Coded Independent Variable Levels**				
	**Corner Points**		**Centre Point**	**Star Points**	
	–1.414	–1.0	0	+1.0	+1.414
*X*_1_ = kafirin (mg)	37.87	100	250	400	462.13
*X*_2_ = prednisolone (mg)	48.25	100	225	350	401.75
Real dependent variables		-			
*Y*_1_ = prednisolone loading (%)		$\frac{\mathrm{amount}\;\mathrm{of}\;\mathrm{prednisolone}\;(\mathrm{mg})\;\mathrm{in}\;\mathrm{microparticles}}{\mathrm{amount}\;\mathrm{of}\;\mathrm{microparticles}\;(\mathrm{mg})}\text{×}100$			
*Y*_2_ = loading efficiency (%)		$\frac{\mathrm{amount}\;\mathrm{of}\;\mathrm{prednisolone}\;(\mathrm{mg})\;\mathrm{in}\;\mathrm{microparticles}}{\mathrm{amount}\;\mathrm{of}\;\mathrm{prednisolone}\;(\mathrm{mg})\;\mathrm{initially}\;\mathrm{added}}\text{×}100$			

**Table 2:** Classification and description of the substrates used for fermentation in the simulated conditions of the colon.

Substrate Number	Substrate Classification	Description
1	Sample: replicate 1	Kafirin microparticles loaded with prednisolone equivalent to 5 mg of prednisolone + fecal inoculum + 0.5 g glucose
2	Sample: replicate 2	Kafirin microparticles loaded with prednisolone equivalent to 5 mg of prednisolone + fecal inoculum + 0.5 g glucose
3	Sample: replicate 3	Kafirin microparticles loaded with prednisolone equivalent to 5 mg of prednisolone + fecal inoculum + 0.5 g glucose
4	Blank	Medium + fecal inoculum
5	Control 1: for glucose	Fecal inoculum + 0.5 g glucose
6	Control 2: for microparticles	Kafirin microparticles (no prednisolone) + fecal inoculum
7	Control 3: for microparticles	Commercially available 5 mg prednisolone tablets + fecal inoculum + 0.5 g glucose
8	Control 4: for microparticles and fecal inoculum	Commercially available 5 mg prednisolone tablets + autoclaved fecal inoculum

**Table 3:** Contributions of independent factors to the mathematical model expressed as coefficients with *p*-values.

Response	Intercept	*X*_1_	*X*_2_	*X*_2_^2^
*Y_1_*	7.25	–0.21	3.37	-
*p*-value	-	0.8301	0.0055	-
*Y_2_*	14.67	5.98	–1.38	5.99
*p*-value	-	0.0417	0.5964	0.0522

NB: Response *Y*_1_ = prednisolone loading (%), *Y*_2_ = loading efficiency (%). The intercept *X*_1_ = linear regression coefficient for the coded quantities of kafirin, *X*_2_ = linear regression coefficient for the coded quantities of prednisolone, while *X*_2_^2^ = the quadratic regression coefficient for the coded quantities of prednisolone.

**Table 4:** Predicted and observed values of the dependent responses for the optimal independent variable combination and a ′non-optimal′ combination.

Dependent variable	Predicted Value	Observed Value	Predicted Error (%)
Optimal independent variable combination: 400 mg kafirin and 350 mg prednisolone			
*Y*_1_ = Prednisolone loading (%)	10.414	13.53	29.9
*Y*_2_ = Loading efficiency (%)	25.269	30.80	21.9
′Non-optimal′ independent variable combination: 200 mg kafirin and 200 mg prednisolone			
*Y*_1_ = Prednisolone loading (%)	6.64	3.73	–43.8
*Y*_2_ = Loading efficiency (%)	13.22	8.33	–37.0

**Table 5:** Particle size analysis of empty and prednisolone loaded DDG kafirin microparticles formulated with 350 mg prednisolone, 400 mg kafirin and 0.1 M NaCl. Data shows the volume mean diameter, *d*, where 10%, 50% and 90% of the particles are below the reported number, the surface area mean diameter D(3,2) and the volume mean diameter D(4,3), all measured in **µ**m.

Microparticles	*d*(v,0.1) (µm)	*d*(v,0.5) (µm)	*d*(v,0.9) (µm)	D(3,2) (µm)	D(4,3) (µm)
Empty	3.317	12.535	33.519	7.632	17.205
Prednisolone loaded	2.534	8.820	34.159	5.946	15.334
